# Editorial: Spectroscopic applications for quality profiling and authentication of food products

**DOI:** 10.3389/fnut.2022.1121385

**Published:** 2022-12-23

**Authors:** R. Pandiselvam, Daniel Cozzolino, Anjineyulu Kothakota

**Affiliations:** ^1^Physiology, Biochemistry, and Post-harvest Technology Division, ICAR-Central Plantation Crops Research Institute, Kasaragod, Kerala, India; ^2^Centre for Nutrition and Food Sciences, Queensland Alliance for Agriculture and Food Innovation (QAAFI), The University of Queensland, Brisbane, QLD, Australia; ^3^Agro-Processing & Technology Division, CSIR-National Institute for Interdisciplinary Science and Technology (NIIST), Trivandrum, Kerala, India

**Keywords:** non-destructive technique (NDT), spectroscopy, chemometric analysis, pre-processed data, spectral response, food authentication technique

Food quality and safety analysis are of paramount importance to ensure public health. Recent changes in eating habits and the modernization of food value chains have demanded the authentication of food ingredients and products. Adulteration in foods and microbiological contamination are two of the main issues affecting public health. The demand for high quality food increases the standards for quality control, which in turns requires high standard analytical tools to ensure the integrity of food products. The conventional analytical techniques are labor intensive, time consuming, and require often toxic chemicals. Food processing industries are looking for a potential tool for rapid, non-destructive, and online testing of quality of foods during the supply chain. Numerous food researchers are exploring the application of spectroscopic techniques for food quality testing and food authentication. Current research and applications of spectroscopic techniques have been reported for quality evaluation of different forms of the foods such as fresh produce (fruits & vegetables), minimally processed foods and/or cut fruits, food treated by non-thermal technologies, modified atmospheric/controlled atmospheric packed food, freeze dried foods, and microwave applied food have recently gained more attention. The food industry, food quality testing laboratories, and food standard organizations are interested in understanding the practical utilizations of spectroscopy techniques for quality evaluation.

This Research Topic provides with recent trend and current information on different spectroscopy methods used for measurement of food quality and safety in the two of the most adulterated food categories: horticulture based fresh and processed food products and animal origin foods including dairy products.

Han et al. evaluated the potential application of Fourier transform near-infrared (FT-NIR) coupled with chemometrics to authenticate animal based blood gels. The samples taken under this study were pig blood-based gel, cow blood-based gel, raw duck blood tofu and its binary and ternary adulterated with cow blood-based gel and pig blood-based gel. Amino acid profile and other biochemical nutrients were determined using FT-NIR. These authors have applied four extreme learning machine regression (ELMR) to predict adulteration levels. Overall, it was found that FT-NIR paired with ELM could able to authenticate the animal blood food with an accuracy of 93.89%.

Authors from India, Romania, Germany and Poland (Pandiselvam et al.) reviewed the recent applications of NIR spectroscopy techniques to assess safety and quality of horticulture products in terms of biochemical quality evaluation, maturity and variety identification, estimation of textural properties and detection of damage and microbial/fungus contamination. These authors highlighted the importance of computational techniques to improve the prediction ability of NIR spectroscopy. Understanding the pre-processing techniques to analyze the spectral data and proper selection of wavelength would help for rapid and non-destructive checking of food quality using NIR spectroscopy.

Chaudhary et al. critically reviewed the authentication of animal origin foods using spectroscopic techniques. In this review, the authors evaluated the efficacy of latest spectroscopic techniques for detection of fraud in fish, meat, egg, poultry, and dairy products. This review highlighted the advantage and disadvantages of each advanced analytical techniques in comparison with traditional/conventional lab techniques.

Qi et al. reported the detection of glutathione in dairy products based on surface-enhanced infrared absorption spectroscopy (SEIRA). The authors have prepared silver nanoparticles as enhancer substrates for the detection of glutathione in dairy products. The collected spectral signatures were analyzed by SEIRA method in transmission mode using a cell of calcium fluoride window sheet immobilization solution. They further analyzed the infrared spectrum peak and the bond interaction for the quantitative analysis of glutathione. Further, they obtained good linearity and correlation coefficients with minimum error value. It confirms that spectroscopy could be used for accurate determination of glutathione content in common dairy products.

We thank the specialty Chief Editor (Food Chemistry) Dr. Michael Rychlik and his team for their support and guidance. We appreciate the authors and reviewers from different countries for their valuable contributions. Our thanks to Frontiers for publishing this Research Topic.

## Author contributions

RP wrote the introduction and the conclusion. RP, DC, and AK wrote the central part with comments to the cited papers. All authors contributed to the article and approved the submitted version.

